# In-silico discovery of dual active molecule to restore synaptic wiring against autism spectrum disorder via HDAC2 and H3R inhibition

**DOI:** 10.1371/journal.pone.0268139

**Published:** 2022-07-25

**Authors:** Anupam Raja, Nishant Shekhar, Harvinder Singh, Ajay Prakash, Bikash Medhi

**Affiliations:** Department of Pharmacology, PGIMER, Chandigarh, India; Jamia Millia Islamia, INDIA

## Abstract

Metal-dependent histone deacetylases (HDACs) are essential epigenetic regulators; their molecular and pharmacological roles in medically critical diseases such as neuropsychiatric disorders, neurodegeneration, and cancer are being studied globally. HDAC2’s differential expression in the central nervous system makes it an appealing therapeutic target for chronic neurological diseases like autism spectrum disorder. In this study, we identified H3R inhibitor molecules that are computationally effective at binding to the HDAC2 metal-coordinated binding site. The study highlights the importance of pitolisant in screening the potential H3R inhibitors by using a hybrid workflow of ligand and receptor-based drug discovery. The screened lead compounds with PubChem SIDs 103179850, 103185945, and 103362074 show viable binding with HDAC2 *in silico*. The importance of ligand contacts with the Zn^2+^ ion in the HDAC2 catalytic site is also discussed and investigated for a significant role in enzyme inhibition. The proposed H3R inhibitors 103179850, 103185945, and 103362074 are estimated as dual-active molecules to block the HDAC2-mediated deacetylation of the EAAT2 gene (SLC1A2) and H3R-mediated synaptic transmission irregularity and are, therefore, open for experimental validation.

## Introduction

Autism spectrum disorder (ASD) is a progressive and lifelong heterogeneous neurodevelopmental abnormality, that can be diagnosed earliest in 2-year-old children [1]. ASD individual shares both the phenotypic and mechanistic features with other neuropsychiatric disorders like Fragile X syndrome(FXS) and intellectual disabilities [IDs] [2–5]; as consequence, it has become clinically so ambiguous and, demographically a common neurodevelopmental disorder [4]. Despite its knotty neurobehavioral repercussions, it is particularly characterized by abnormal social interactions, eye contact, social communications, and stereotypic behavior with conditional interests and activities [6, 7]. However, ASD individual also shows neurochemical signaling alteration similar to anxiety, intellectual disability, seizures, epilepsy, attention, motor abnormalities, language deficits, sleep disturbance, hyper or hypo-reactivity, and gastrointestinal problems [8–11]. There are several explanations for the pathophysiology that is associated with ASD, like alteration in glutamatergic, GABAergic, dopaminergic, serotonergic, cholinergic, and histaminergic signaling [9, 11, 12]. Glutamatergic and histaminergic signaling are the majorly involved (according to postmortem reports) and, most explored wiring abnormalities hitherto, which are associated with ASD patients [13, 14]. Moreover, glutamate transporters comprise five sub-types, EAAT-1,2,3,4 and 5, which play a significant role in maintaining the physiological level of glutamate during synaptic transmission. Synaptic glutamate (~90%) is transported into astrocytic cells, particularly by the glutamate transporters subtype EAAT2. Interestingly, reduced EAAT2 mRNA and protein level has been found in the autopsy of the autistic brain. In addition, EAAT2 expression in astrocytic cells can be upregulated or downregulated at the transcriptional level by different modulators [15]. Out of eleven isoforms of HDAC (Histone deacetylase), particularly HDAC2 is responsible to regulate the mRNA and protein level of EAAT2 and, regulating the synaptic plasticity which results in amelioration of behavioral and cognitive abnormalities [16]. Furthermore, atypical histaminergic wiring can alter several physiological balances such as neurochemical, neurobiological, and hormonal homeostasis [17]. As a consequence, it is becoming the key contributor to neuropsychiatric disorders like ASD, FXS, Dementia, and schizophrenia [18]. Histamine H3 receptor (H3R) is a subtype of histamine receptor (HR), which has relatively elevated expression levels in the brain and, acts as an auto-receptor and heteroreceptor (because it regulates the release of other neurotransmitters like ACh, Glu, GABA, 5-HT, and DA) [17]. Interestingly, antagonizing the H3R (e.g., famotidine) has been found to have a possible therapeutic role in managing autistic-like symptoms [19]. Considering the mechanistic complexity of this particular disease, it is necessary to look multiple molecular targeted therapies.

**Fig 1:**
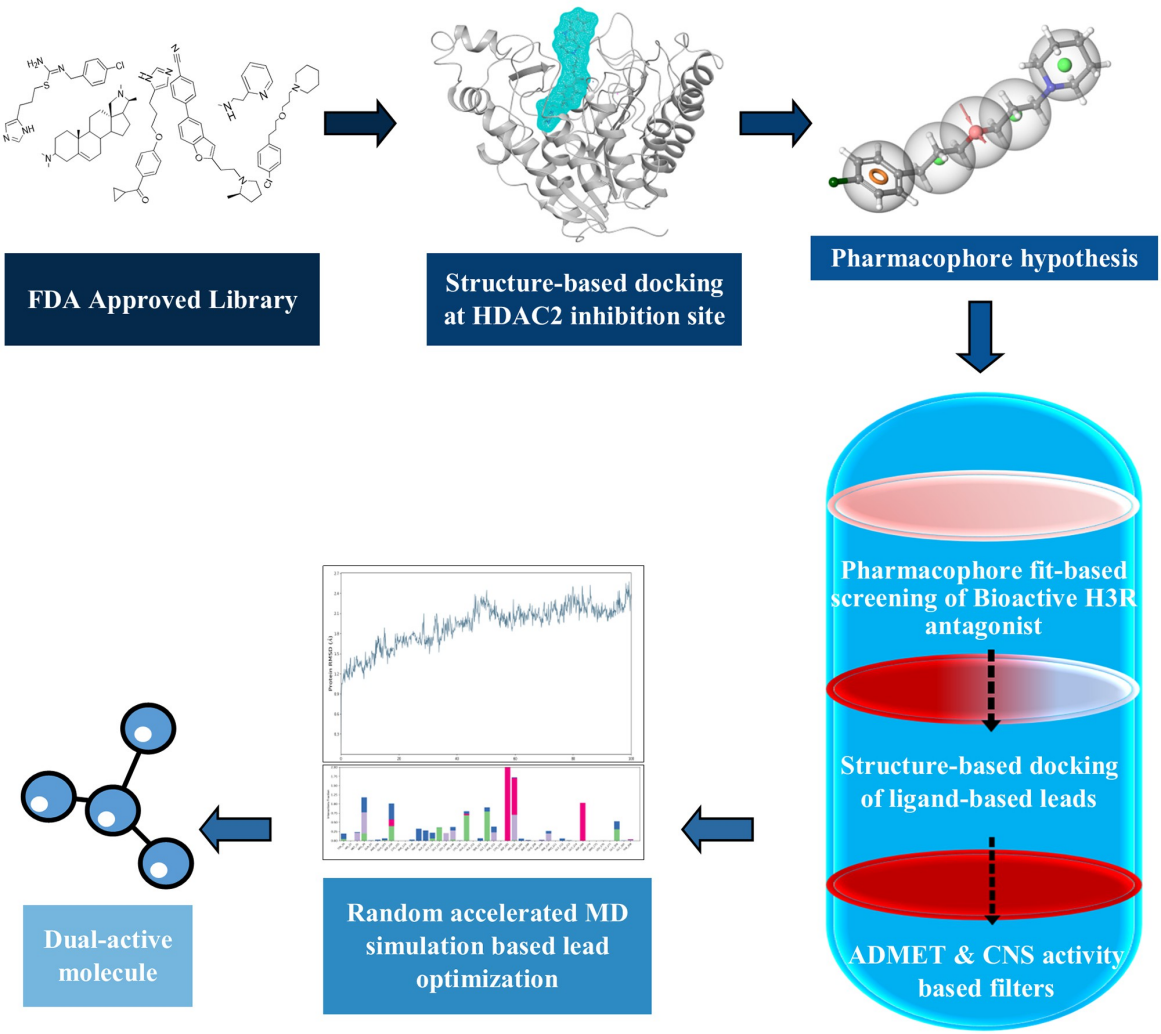
Workflow of the methodology; the progressive flow chart describes the steps involved in the screening of potential HDAC2 binders starting from H3R reported inhibitors.

In the present study, we intend to screen and optimize the FDA-approved H3R inhibitor molecules that exhibit computationally potent binding on the HDAC2 binding site. The strategic drug optimization involves the selection of reported H3R antagonists to perform molecular docking and binding free energy-based analyses for the selection of an ideal molecule to produce an e-pharmacophore model. The following screening was based on a series of observations, beginning with the pharmacophore fit score of the candidate library, moving on to molecular docking, and finally to MD simulation-based trajectory analyses of the top-scoring hits. A brief illustration of the workflow can be seen in Fig 1.

## Material and methods

### 1. Protein and ligand structure modeling

The protein crystal structure of HDAC2 was acquired from https://www.rcsb.org/ with the PDB id 5IX0 [20]. The HDAC2 molecule in this PDB model represents 131.25 kDa molecular weight with a sequence length of 369 amino acids. An inhibitor molecule, 6EZ also comes co-crystallized with this structure. The refinement and preparation of this protein were done with the aid of PrepWiz from Schrodinger [21]. Additional ligands excluding the co-factor Zn^2+^ and Ca^2+^ ions were deleted from the crystal structure, the 6EZ molecule was left co-crystallized with the HDAC2 inhibition site for coordinate information required in receptor grid generation. H-bonds were optimized according to pKa = 7.2 and restrained minimization was performed with an OPLS3 force field [22]. OPLS3 maintains a high degree of precision through consistency benchmarks, assessing the conformational propensities and solvation of small molecules relative to the MMFF and OPLS_2005 force fields. The receptor grid at the HDAC2 inhibition site was generated using GLIDE [23, 24]. The coordinate information of 6EZ was utilized to specify the receptor cavity centroid of the HDAC2 inhibition site, the output files were utilized for molecular docking.

H3R modeling was performed on the Robetta server by Baker Lab using the trRosetta method with the input protein sequence of NCBI accession number NP_009163.2 [25]. trRosetta uses a deep learning-based modeling method, which uses a deep network for the prediction, in addition to distances, inter-residual orientations, and a protocol for the restricted Rosetta energy minimization for the fast and precise production of structure models that are driven by the above-mentioned constraints. The validation of structural stability was assessed from the attributes produced by MD simulations, particularly cross-validating the ligand-protein residue specificity of reported H3R inhibitors.

The ligand molecules were acquired from Drugbank (FDA approved library) and individually from PubChem published bioassays of H3R inhibitors in SDFSDF format [26, 27]. LigPrep module was utilized to create ligand 3D structures at target pH = 7.2 +/- 2.0, LigPrep is a Schrodinger Maestro tool specialized in generating accurate and energy minimized 3D structures with a wide diversity of electrochemical state generation of ligand molecules [28].

### 2. Molecular docking and virtual screening

For the structure-based drug discovery study, the GLIDE module from Schrodinger was used, the effective tandem molecular docking scheme provided under the tool virtual screen workflow allows for the convenience to acquire a set of high-affinity molecules. The scoring functions used in exhaustive screening are SP (standard precision) and XP (extra precision) docking. The screening specificity in terms of distinguishing the false positive hits (decoys) from true positive hits (actives) was assessed via enrichment analysis with ROC and AUC attributes of the screen output file. The benchmark decoy and hit libraries for HDAC2 protein were obtained from the DUD-E database [29]. There was a total of 238 actives, the number of decoys deployed for the enrichment analysis was 1000, making it a total of 1238 distinct molecules, ruling out the multiple conformer generation to before the enrichment.

The final scoring of the virtual screening was achieved using the MM-GBSA binding free energies of the receptor-ligand complexes. The MM-GBSA elicitation tool is provided under the module name Prime of Schrodinger Maestro [30]. The solvation model used in this technique is VSGB 2.0, which estimates the solvation free energy with a typical Generalized Born model and variable dielectric constants of polar side chains in the amino acids [31]. The protein flexibility parameter was defined for residues within 5.0Å of the ligand-binding site. The following assessment was done for the top-scoring hits according to the trend that lower (more negative) ΔG_bind_ values infer high binding affinity.

### 3. Pharmacokinetic assessment of the hits

The pharmacokinetic evaluations for the hits with high binding affinity were done with the help of publicly open servers SwissADME, admetSAR and, Qikprop tool from Schrodinger [32–34]. A particular emphasis was given to the blood-brain permeability and Central Nervous System (CNS)activity in addition to drug-likeliness, human oral absorption, sub-cellular specificity, and safety of the screened hits, so as to regress the rationale of screening potent and safe neuroactive molecules. The additional element that we complied was the fact that cells with H3R and native HDAC2 are of neurological origin. The most suitable hits were then carried forward for random accelerated molecular dynamics (RAMD) simulations-based analysis.

### 4. MD simulations and trajectory analysis

All the full-scale molecular dynamics simulations were run on Desmond from Schrodinger [30]. The solvent system for all simulations was constructed using the TIP3P water model within orthorhombic boundary conditions, and neutralization of the systems was done with counter ions to ensure the total charge becomes zero. The system was minimized using the hybrid algorithm of the steepest descent method and the limited memory Broyden-Fletcher-Goldfarb-Shanno (LBFGS) algorithm for 100ps prior to MD simulation [35]. 100ns simulations were run with Nose-Hoover thermostat NPT (constant composition, pressure, and temperature)ensemble at 310 K temperature and 1.01325 bar pressure, where velocities were randomized at every 1ns interval [36]. The binding free energies of the protein-ligand complexes were calculated using thermal MM-GBSA python script provided by Schrodinger, on the MD trajectory’s ensembles of protein-ligand complexes at 1ns interval. The Prime module expresses the binding free energy as the summation of potential energies (PE) of the system [37, 38]:

$$\Delta {\text{G}}_{\text{bind}}={\text{PE}}_{\text{complex}}-{\text{PE}}_{\text{free}\;\text{ligand}}-{\text{PE}}_{\text{freeprotein}}$$

The ΔG_bind_ calculations of protein-ligand complexes from a MD trajectory can provide insights on the relative affinities of ligand molecules describing the retention and attributes of the occupancy of the ligand in the binding site. The simulation interaction data extraction and trajectory analyses were performed using Desmond, VMD, Bio3D, and Rstudio [39–41]. The reference frames of 0 ns were selected for each complex; hence, the relative deviations were recorded in accordance with the conformational states at 0 ns.

## Results

### 1. The structure of HDAC2 and histamine H3R receptor

**Fig 2:**
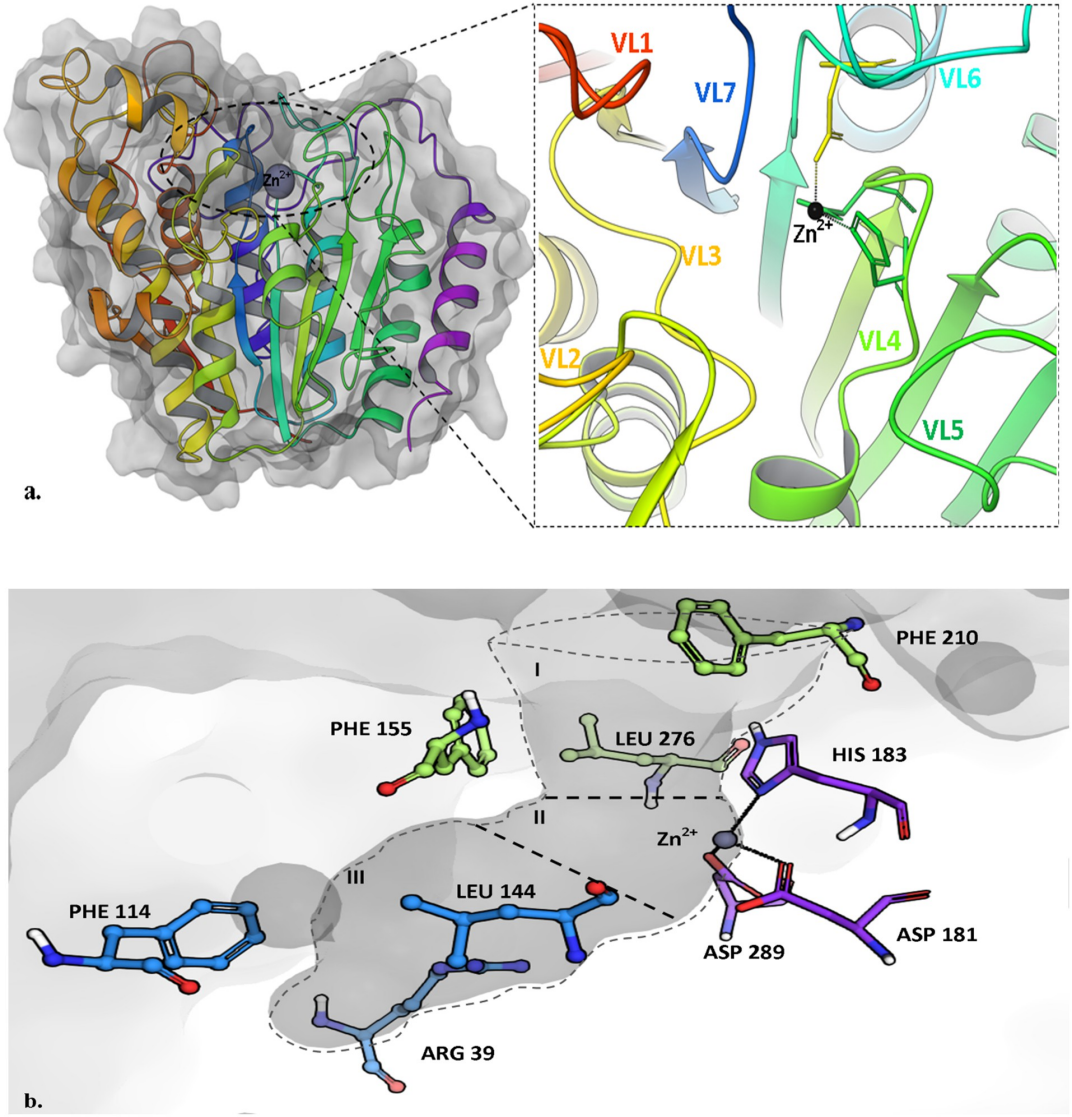
Structure of HDAC2. (A) Cartoon representation of HDAC2 highlighting the variable loop region making the orifice of the binding cleft. (B) HDAC2 binding site featuring the active site residues in 3 regions; (I) hydrophobic pit, (II) metal-coordinated catalytic pocket, and (III) foot pocket.

Human metal-dependent histone deacetylases (HDAC), based on phylogenetic heterogeneity and functional characteristics, can be characterized into 5 distinct classes, i.e., HDAC1, 2, 3, 8 (class I), HDAC4, 5, 7, 9 (class IIa), HDAC6, 10 (class IIb), the sirtuins SIRT1–7 (class III), and HDAC11 (class IV) [42]. The structural and biochemical description of the HDAC2 active site particularly can be summarized as a narrow pit-like invagination at the center of the multiple loops, and a Zn^2+^ ion occupying the lower bottom of the pit. The difference in the substrate and antagonist specificity among different HDAC subtypes arises due to the variation in the architecture of the surrounding secondary structure of the binding site. The binding site of HDAC2 in Fig 2 is understood effectively if it is structurally divided into 3 portions i.e., a hydrophobic tube (or pit), the catalytic pocket, and foot pocket. The catalytic pocket is occupied by Zn^2+^ bound to Asp 181, His 183, Asp 269 via metal-coordinate bonds, this pocket is bridged to the surface pore through the tube-like hydrophobic invagination (Gly154, Phe155, Phe210, and Leu276) and just adjacent to the catalytic site is the foot pocket (Arg39, Met35, Phe114, and Leu144) which provides anchorage to the polar substrate or inhibitor.

**Fig 3:**
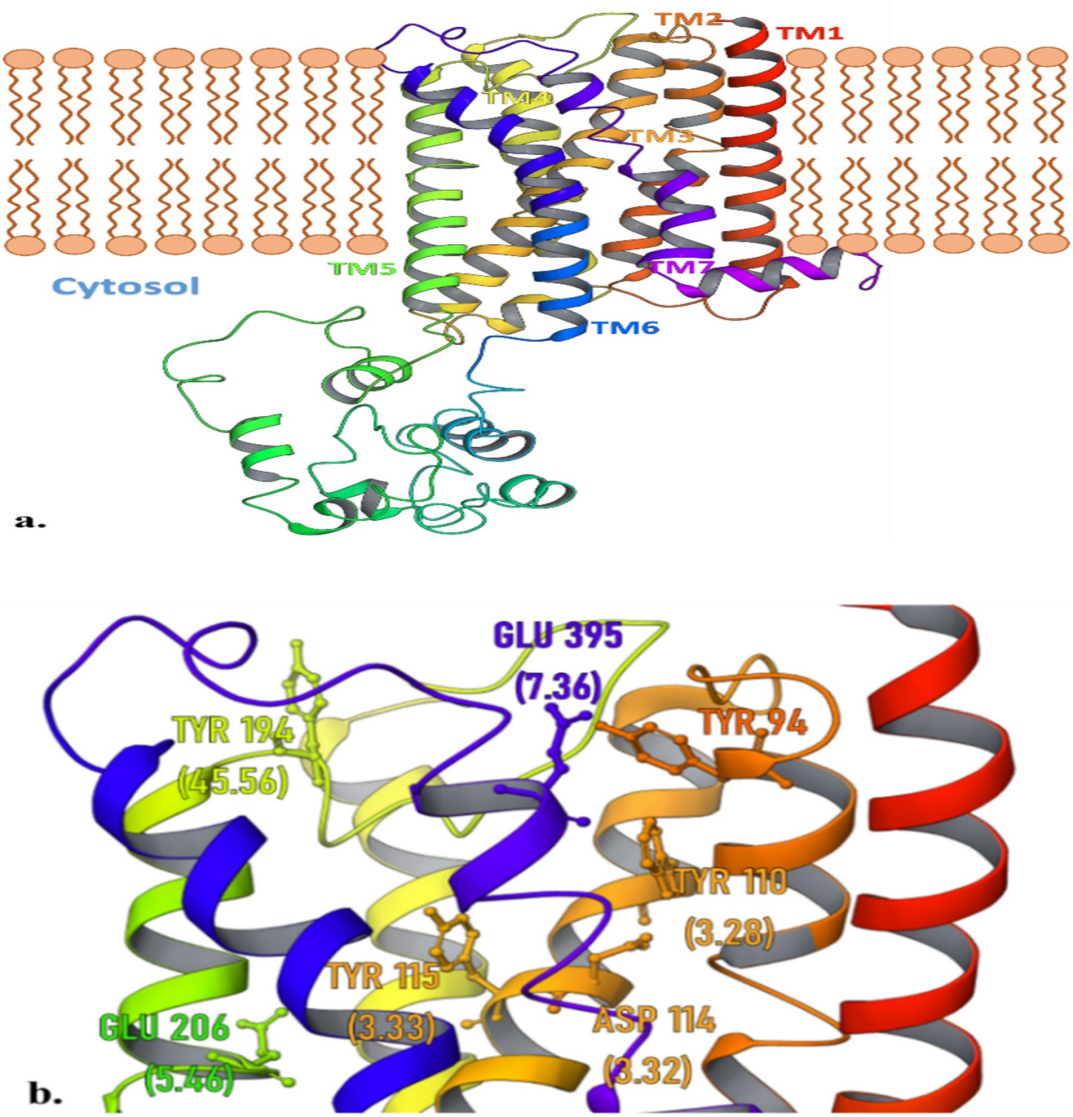
tRosetta modelled structure of human H3R receptor. (A) The H3R is featured using successive transmembrane (TM) domains; the big cytosolic lobe is associated with the effector activity with G-protein. (B) Catalytically active residues of H3R take part in agonist and antagonist binding.

The human histamine H3 receptor concerning its other subtypes is expressed highly in nervous tissues. Being a serpentine GPCR, its N-terminal and C-terminal ends are exposed to extracellular and intracellular interfaces respectively. The sequence alignment of H3R using blastp algorithmic search on protein data bank (PDB) database ascertains that histamine H3R shares maximum homology with M1 Muscarinic acetylcholine receptor [6OIJ] (% identity = 38.92), while the histamine receptor subtype H1R [3RZE] shares 25.98% similarity. There have been several studies that utilized the homology-based modelled structure of H3R and illustrated the binding of H3R inhibitors providing a knowledge-based estimation of its active site residues [43, 44]. The trRosetta output model shown in Fig 3A represents the trans-membranous 3D model of histamine H3R molecule, where the consecutive transmembrane domains are highlighted distinctively in Fig 3B encircling the histamine binding site. This site is primarily characterized by Asp 114 (3.32), Trp 110(3.28), Trp 291(7.43), and Phe 192(45.54) particularly for histamine binding, and are also shared by reported antagonists e.g. ciproxifan, thioperamide, pitolisant, and ABT 239 [43, 45]. We intend to study the analogy between the binding site features of HDAC2 and H3R receptor which here is executed using the hybrid approach of structure and ligand-based computational tools.

### 2. e-Pharmacophore features of Pitolisant and its selectivity for HDAC2

**Fig 4:**
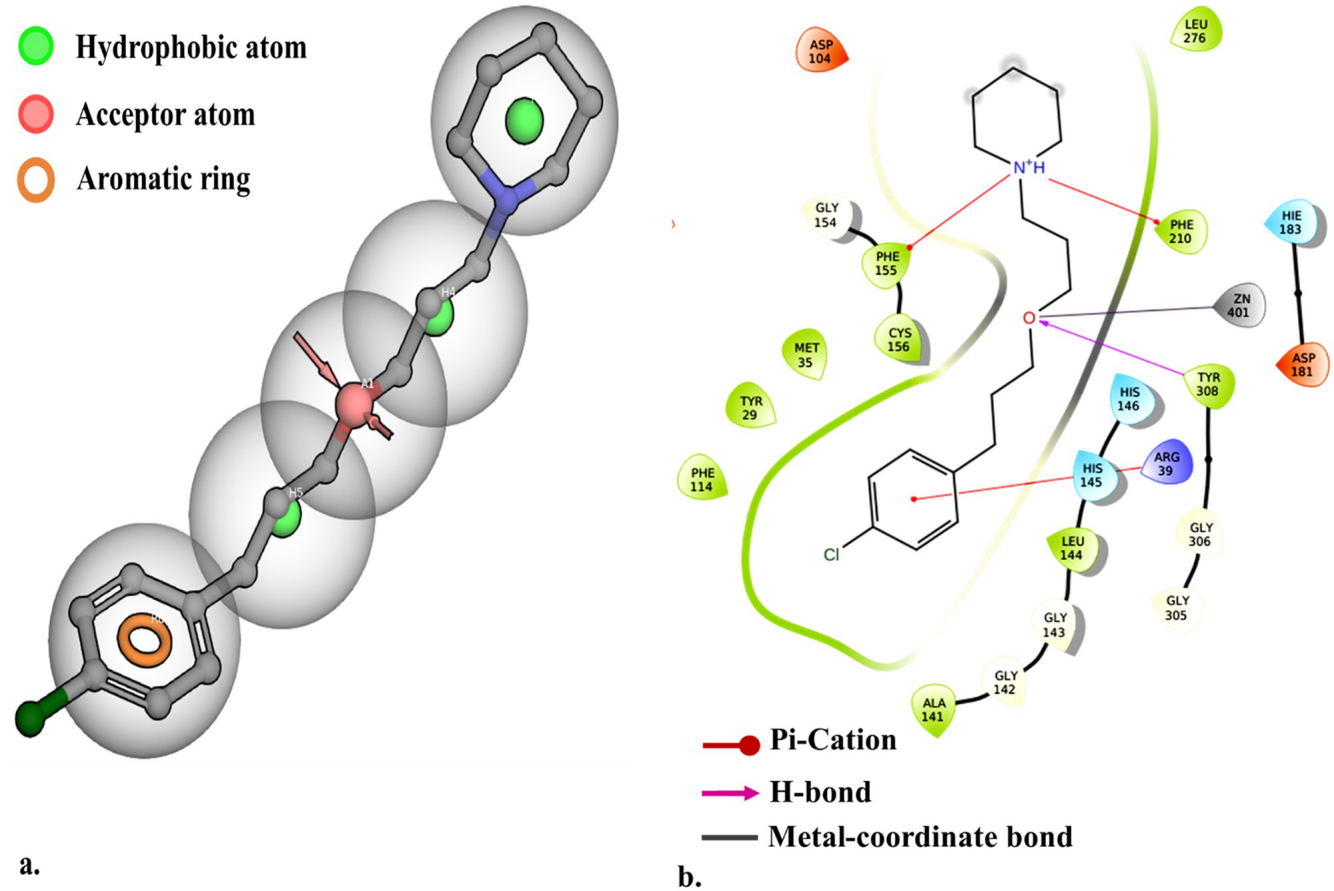
(A) Pharmacophore hypothesis using pitolisant as a model molecule and, (B) 2D interaction diagram of molecular docking of pitolisant at HDAC2 binding site.

The receptor-based molecular docking of biochemically and clinically established H3R inhibitors/inverse agonists (Table 1) at the HDAC2 inhibition site highlights the comparative selectivity of HDAC2 active residues for these molecules, which is further described by their computed MM-GBSA ΔG_bind_ and experimental Ki values. It is evident that pitolisant exhibits highly favorable binding energy and docking scores with HDAC2, however, the binding of clobenpropit and ciproxifan is also commensurable with pitolisant in contrast to the following H3R antagonists. Comparing the molecular features of pitolisant with the previously described pharmacophore model of HDAC2 inhibitors, as in Miller et al. 2003, pitolisant delivers a good fit [46]. Pitolisant or 1-(3-(3- [4-chlorophenyl]propoxy)propyl)piperidine hydrochloride can be structurally described as a piperidine-ring (a hydrophobic ring with solvable hydrogens), connected to chlorobenzene (an aromatic ring with a hydrophobic group), via ether linkage (a hydrophobic linker chain with acceptor oxygen). These features of pitolisant can be visualized as a pharmacophore hypothesis in Fig 4A. The piperidine ring acts as the cap at the pit’s orifice, exposed to the solvent phase. The amine (-NH^+^-) group of the piperidine ring provides electrostatic anchorage with the Phenylalanine residues (Phe 155 & Phe 210) by Pi-cation interaction. The ether link of pitolisant expresses fair hydrophobic interaction with the pit’s tube and its -oxy- group is chelated by a metal-coordination bond with Zn^2+^. In addition, a strong H-bond is seen between the -oxy- group and Tyr 308. The foot pocket of HDAC2 is occupied by the chloro-benzene group and fits well with the ideal HDAC2 antagonist pharmacophore owing to its aromatic ring structure Fig 4B. Nonetheless, clobenpropit and ciproxifan also share these features but not all, and, pitolisant, which is FDA approved for neurological disorders, aids in the selection of neuroactive leads.

**Table 1:** Binding profile of experimentally validated H3R inhibitors against HDAC2 active site.

S no.	(Canonical Smile) Compound	K_i_(μM)	GLIDE docking score	MM-GBSA ΔG_bind (kcal mol_^-1^)
			Interaction with HDAC2	
**1**	C1CCN(CC1)CCCOCCCC2 = CC = C(C = C2)Cl Pitolisant	0.0003 [HRH3 - histamine receptor H3 (human)]	-10.949	-87.81
**2**	C1 = CC (= CC = C1CN = C(N)SCCCC2 = CN = CN2)Cl Clobenpropit	0.0001 [HRH3 - histamine receptor H3 (human)]	-7.814	-86.84
**3**	C1CC1C (= O)C2 = CC = C(C = C2)OCCCC3 = CN = CN3 Ciproxifan	0.0005 [Hrh3 - histamine receptor H3 (Norway rat)]	-7.429	-81.18
**4**	CC1CCCN1CCC2 = CC3 = C(O2)C = CC (= C3)C4 = CC = C(C = C4)C#N ABT-239	0.0004 [HRH3 - histamine receptor H3 (human)]	-3.634	-67.83
**5**	CC1C2CCC3C2(CCC4C3CC = C5C4(CCC(C5)N(C)C)C)CN1C Conessine	0.0050 [HRH3 - histamine receptor H3 (human)]	-2.697	-57.81
**6**	CC(C(C(C(COC (= O)C)OC (= O)C)OC)OC)OC (= O)C A349821	0.00027 [HRH1 - histamine receptor H1 (human)]	-0.041	-52.02
**7**	CNCCC1 = CC = CC = N1 Betahistine	2.03 [Hrh3 - histamine receptor H3 (Norway rat)]	-5.055	-36.01

### 3. Virtual screening and lead optimization

Ligand-based screening of the H3R antagonist using pitolisant as pharmacophore yielded output molecules on the basis of pharmacophore feature homology. High homology correlates with similar structural skeleton and bond lengths as in pitolisant. The Phase fitness score is narrated as a linear combination of the site score, vector alignment scores and the volume fit score. The top 75% of outputs of these were selected from this method and were further employed for structure-based discovery.

**Fig 5:**
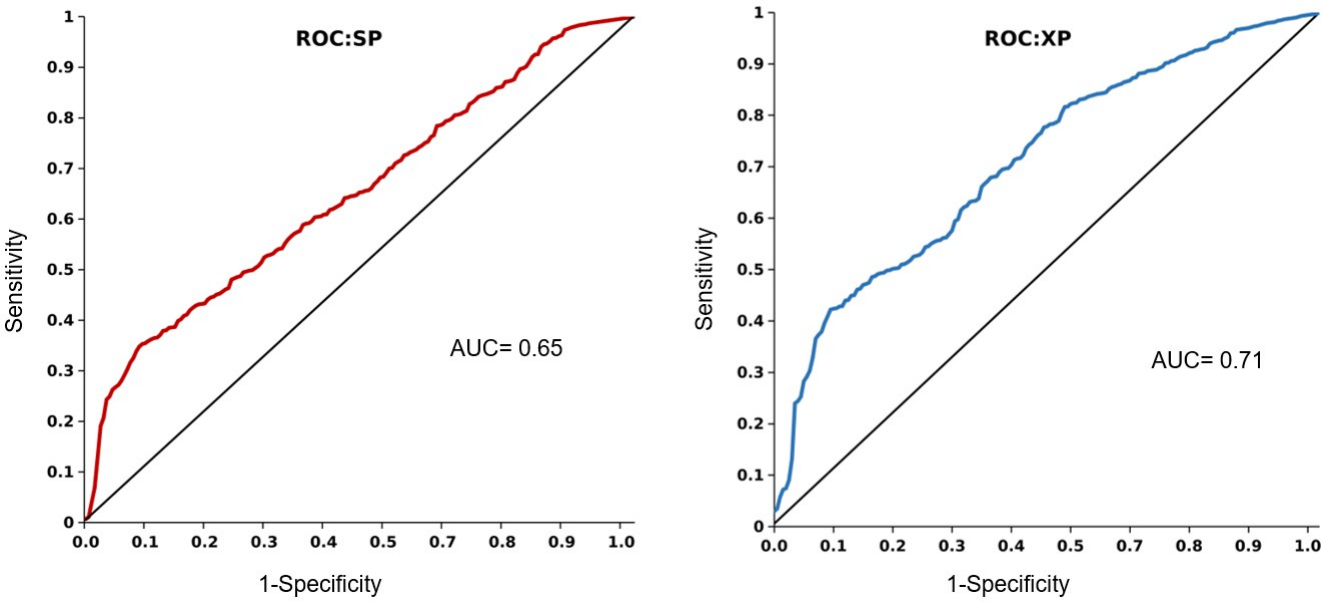
Docking enrichment in GLIDE SP and XP mode against HDAC2. The AUC value describes the ability to discriminate the number of screened actives out of the pool of random decoys and actives. (AUC value ≥ 0.68→ Fair).

The Enrichment analysis of the molecular docking using GLIDE scoring functions, Standard Precision (SP) and Extra Precision (XP) characterizes the distinguishability of screening out the false positives and true positives as ranked outputs. The Receiver operating characteristic (ROC) curve shown in Fig 5 displays this distinguishability score in terms of the Area Under accumulation Curve (AUC) for SP and XP docking. This enrichment of docking procedure varies with different receptor proteins and the actives-decoys selection; hence it provides validation on the complete model and the tools utilized in a study for molecular docking study. The calculated ROC descriptors for XP and SP were 0.75 and 0.67 respectively and, out of 185 total actives, the output actives in the top 10% screen result of 1185 total candidates were found equal to 53 and 37 respectively. These figures classify the SP and XP docking modes as fair to good classifiers.

The final scoring of HDAC2-targeted docking was carried using XP docking mode. This screening output was reevaluated using MM-GBSA ΔG_bind_ values for relatively precise binding affinity estimation. Out of the total 334 experimental H3R inhibitors, 11 were selected on the structure-based evaluation, which makes the top 3.3% of the total screen output. Comparing this value with the enrichment analyses, the number of actives found in the top 2% and 5% of the total hits (1185) were 17 and 32 respectively. Consequently, the probability of finding an HDAC2 active lead within the experimental candidates must be within the range of 0.54 to 0.71. However, the wide range of these candidates in terms of the binding attributes persuaded us to assess their estimated ADME properties, neuroactivity, and toxicity profile. It can be effortlessly assumed that the candidate compounds in Table 2 exhibit viable drug-likeliness and except 103173476, the top ten candidates show BBB permeability which in certain measures is attributed to their respective high logP values. However, the selection of leads on the basis of ADME profile alone was ambiguous owing to the positive but weak CNS active scores of few compounds and lesser availability of the information describing their toxicity profile. Hence, MD simulation-based results were adopted to derive the relationship between the molecular aspects on their interaction with HDAC2 and possible inhibitory mechanisms.

**Table 2:** Estimated ADME properties, neuroactivity, and toxicity profile of screened leads.

PubChem substance ID	SwissADME				Qikprop		admetSAR							Ki (uM) Against H3R
	Mol wt.	Consensus Log P	GI absorption	# Lipinski violations	QlogS	CNS activity	Blood- Brain Barrier	Carcinogenicity (binary)	Eye irritation	Hepatotoxicity	Human oral bioavailability	Pgp inhibitor	Plasma protein binding	
**103179850**	441.5	2.09	High	0	-6.83	-2	+	-	-	+	-	+	0.44	0.05
**103185945**	399.25	3.37	High	0	-5.8	-1	+	-	-	-	-	-	0.78	0.041
**103360761**	366.54	2.17	High	0	-3.93	2	+	-	-	-	+	+	1.01	0.001
**103360711**	365.53	3.27	High	0	-3.96	2	+	-	-	-	+	+	0.9	0.008
**103361521**	316.5	3.93	High	0	-5.8	1	+	-	+	-	-	+	0.88	0.050
**103360810**	360.51	1.48	High	0	-1.46	1	+	-	-	-	-	+	0.71	0.0009
**103360811**	332.5	1.72	Low	0	-0.85	2	+	-	-	-	-	+	0.64	0.001
**103360819**	319.46	1.76	High	0	-1.3	2	+	-	+	-	+	+	0.76	0.0005
**103362074**	461.55	3.27	High	0	-6.03	0	+	-	-	+	-	+	0.87	0.41
**103360818**	331.52	3.04	High	0	-3.56	2	+	-	-	-	+	+	0.77	0.0001

### 4. The structural dynamics of HDAC2 inhibition

**Fig 6:**
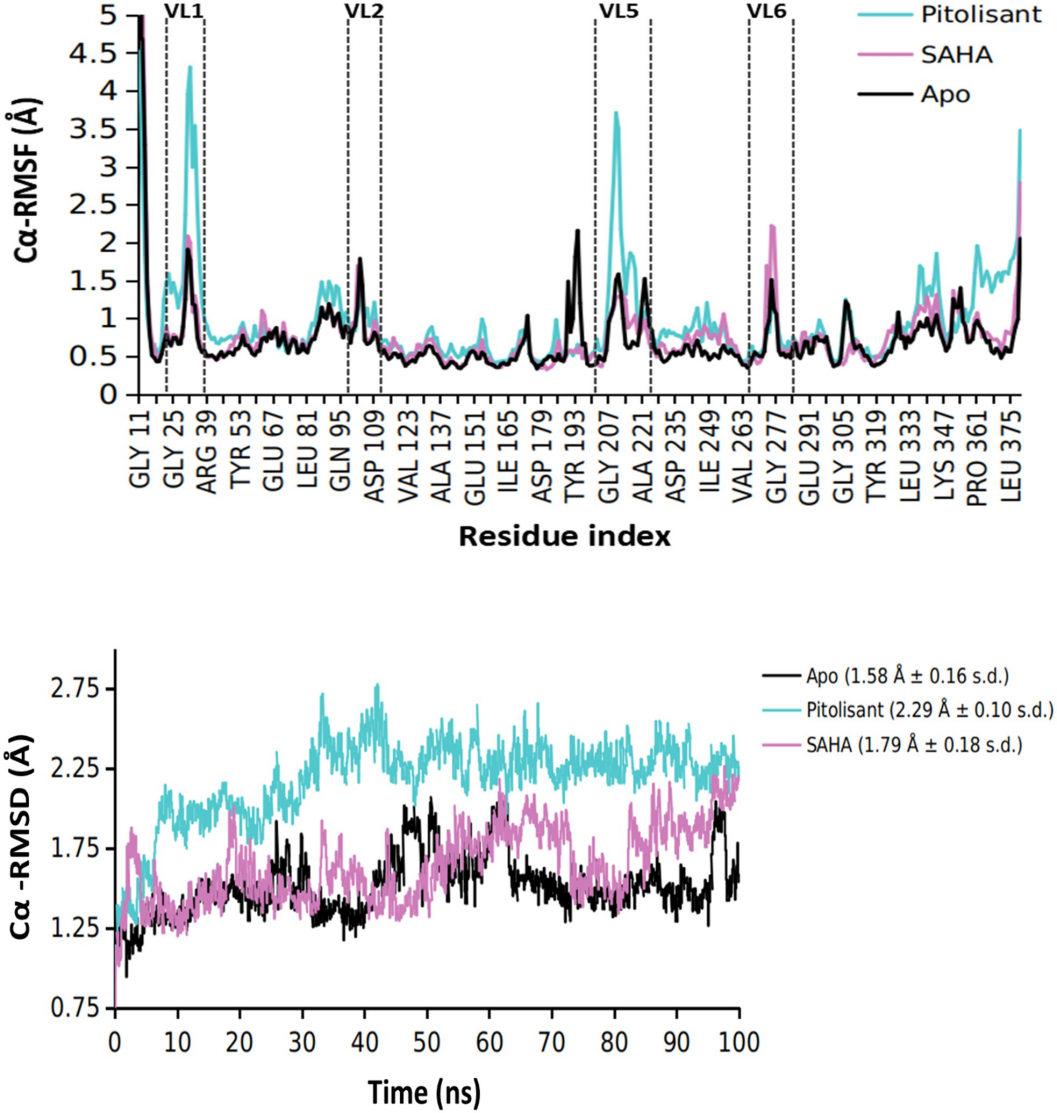
MD simulation profile of Apo (unbound HDAC2), pitolisant-bound and SAHA-bound HDAC2. (A) Root mean square fluctuations (RMSF) and (B) Root mean square deviation of protein Cα carbon during the simulation duration (100 nanoseconds).

**Fig 7:**
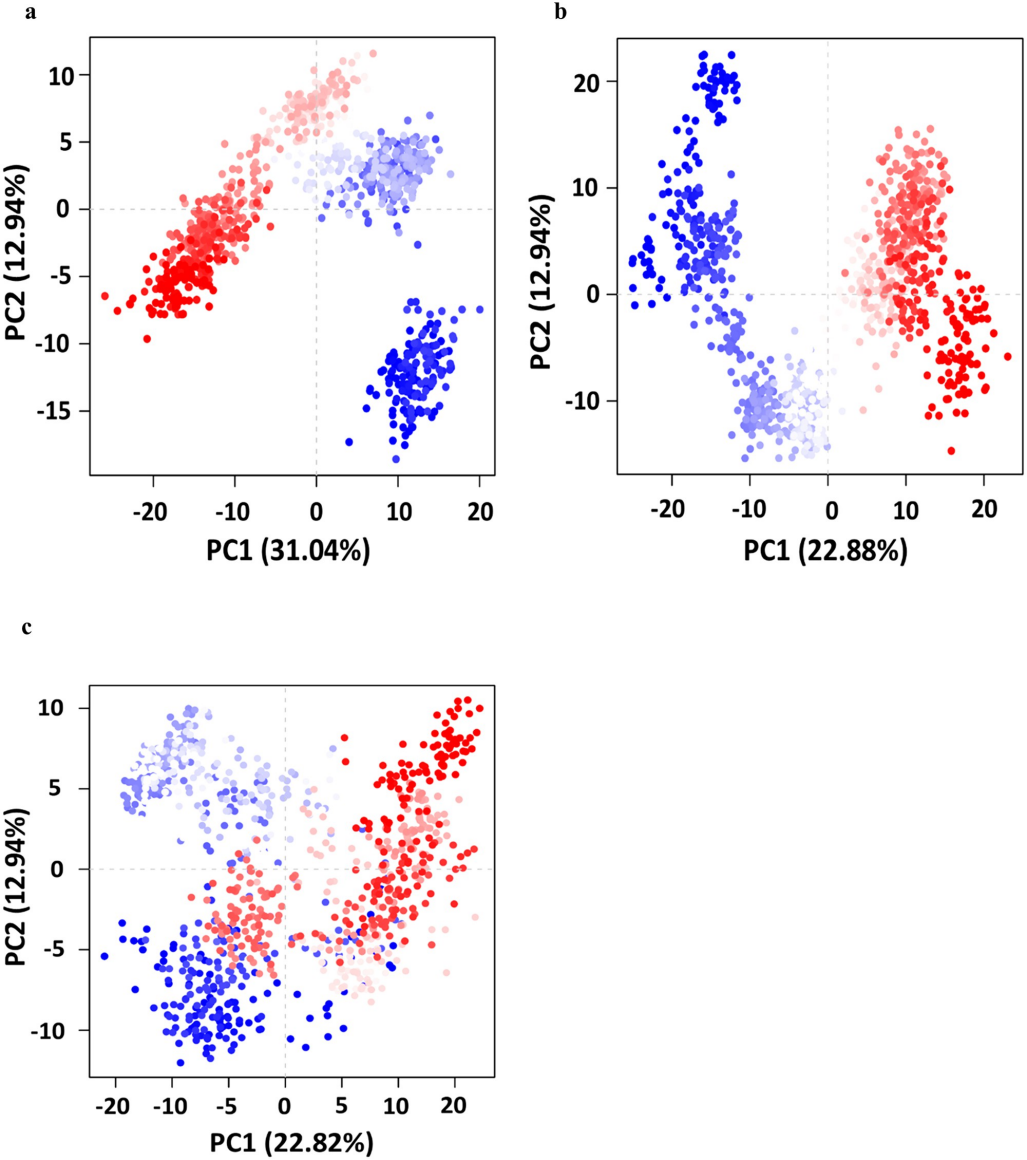
Principal component analysis (PCA) plots for HDAC2 conformations in apo, pitolisant-bound and SAHA-bound trajectory frames (coloured from blue to red in order of time). PC1 and PC2 collectively account for first 2 eigenvalue ranks with overall variations of 43.98% (Apo), 35.82% (Pitolisant) and 35.76% (SAHA) in HDAC2 structure.

The structural basis for HDAC2 inhibition from a perspective of computational modeling can be explained in terms of the ligand-target binding strength and binding site occupancy. Comparative exploration of the variations in crucial domains of HDAC2 concerning time reveals energetically stable and unstable regions. A free (unbound) HDAC2 molecule experiences significant fluctuations in the variable loop regions Fig 6A, which makes up the orifice of the metal-dependent substrate binding site of HDAC2. The overall RMSD of apo HDAC2 was 1.58 Å ± 0.16 s.d. The fluctuations in these variable loops were observed decreasing for HDAC2 complexed with pitolisant and SAHA, while pitolisant also displays higher fluctuations of the variable loop (VL) 1 and 5. The binding of SAHA is exceptionally strong with the HDAC2, which is also attributed by the strong chelation of carbonyl group with Zn^2+^, hence anchored strongly by Asp 181 and His 183. The occupancy of SAHA in the foot pocket was seen with Arg 39, Trp 140, and Gly 142. Moreover, the overall ligand RMSF for SAHA was <0.5 Å, hence stable ligand binding can be ascribed. Nevertheless, pitolisant shows around 60% interaction strength, mostly hydrophobic and with weak Zn^2+^ chelation, this was accompanied with higher ligand fluctuations (~1.4 Å). There was, however, strong Pi-cation interaction with the amide group of pitolisant to His 183 and His 146 and, it stabilized along the simulation length. In addition, no equilibrated protein form converged to a steadier complex in any of the above cases which is evident by the C_α_ RMSD Fig 6B. These conjectures provided by the MD simulations make it more crucial to investigate the experimental leads and assess their binding profile. The spread of conformational spaces, depicted by the PCA plots in Fig 7 clearly describes the relatively high frequency of different conformations obtained by the Apo form. On the other hand, ligand binding has significantly reduced the number of eigenvectors and hence the spread of conformational spaces, describing the constrained activity of HDAC2 in ligand-bound states.

### 5. Comparative dynamic ligand binding reveals strong leads of HDAC2 inhibition

**Fig 8:**
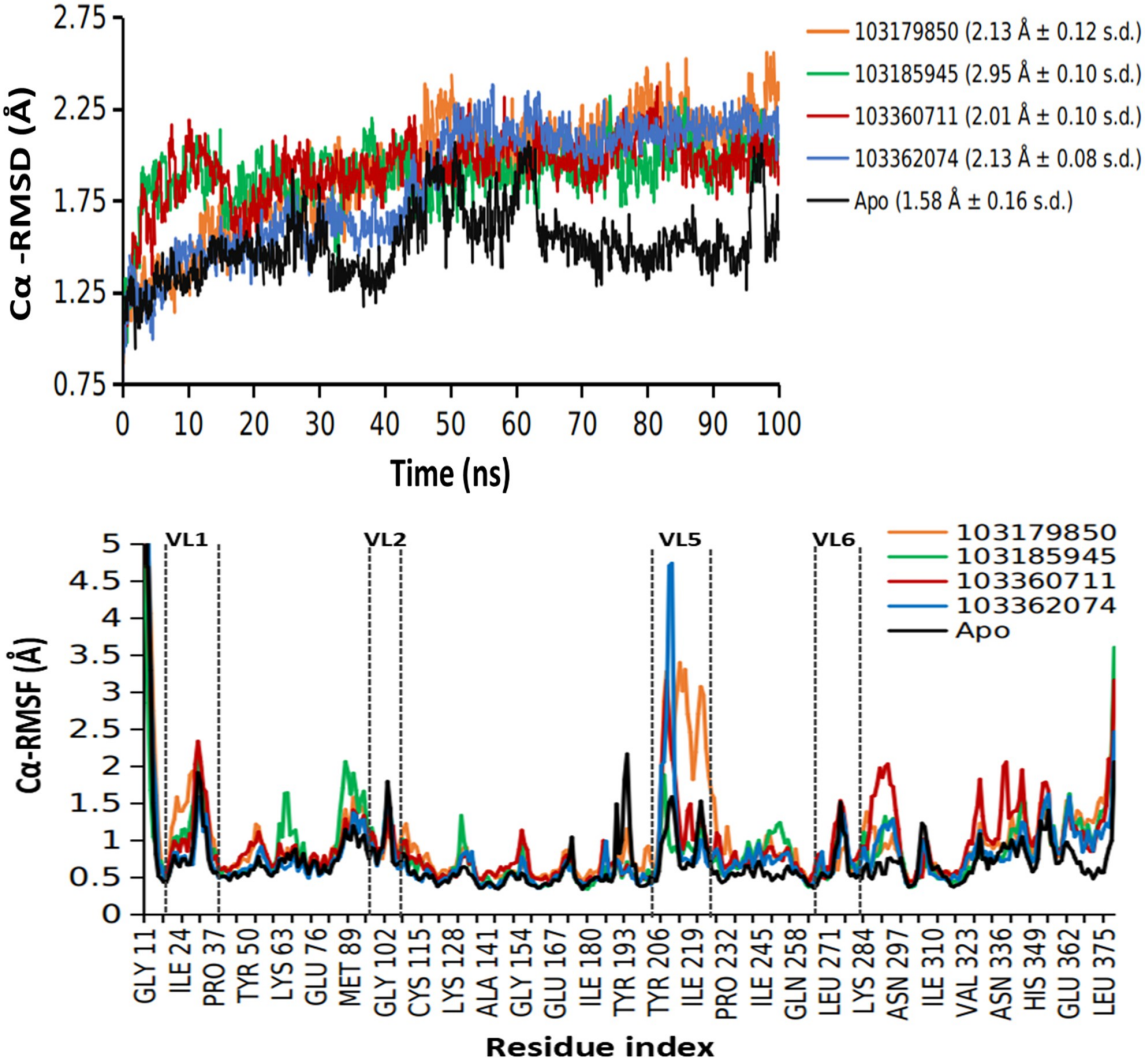
MD simulation profile of Apo (unbound HDAC2), 103179850, 103185945 and 103362074-bound HDAC2. (A) Root mean square fluctuations (RMSF) and (B) Root mean square deviation of protein Cα carbon during the simulation duration (100 nanoseconds).

The quest is to identify potent H3R inhibitors with significant interaction properties with the HDAC2 binding site, also surpassing the binding attributes of pitolisant and possibly resembling SAHA. MD simulations of the selected H3R inhibitors revealed four molecules with good binding profiles, a cut above pitolisant but still, exhibiting all the above-mentioned pharmacophore features. A series of analyses led to the identification of compounds 103179850, 103185945, and 103362074 as substantially good ligands. Extensive observations of the protein-ligand trajectory frames provided insights on the HDAC2 active site flexibility and the affinity of the ligand. Protein-ligand complex stability was primarily investigated by studying theC_α_ RMSD and RMSF of ligand-bound HDAC2. Fig 8A delineates the trendline of ligand-bound RMSDs and it is apparent from the plot, that the equilibration of protein-ligand complexes commences at around 50ns to 100ns, the averages of RMSD in the plot represent the same duration. The least deviated complex was HDAC2 bound with compound 103185945 (1.95 Å ± 0.11 s.d.) and the steadiest RMSD timeline was observed for the 103360761-bound HDAC2 molecule (2.13 Å ± 0.08 s.d.). Compared with the apo, pitolisant-bound, and SAHA-bound forms, the candidate protein-ligand complexes have converged after equilibration after 50 ns, suggesting more readily forming protein-ligand complexes. Given that the differences in average RMSD values among these candidates are not highly significant (range: 1.95 to 2.13 Å), the local variations brought about by these candidates in the HDAC2 proteins provide an explicit overview on ligand binding.

**Fig 9:**
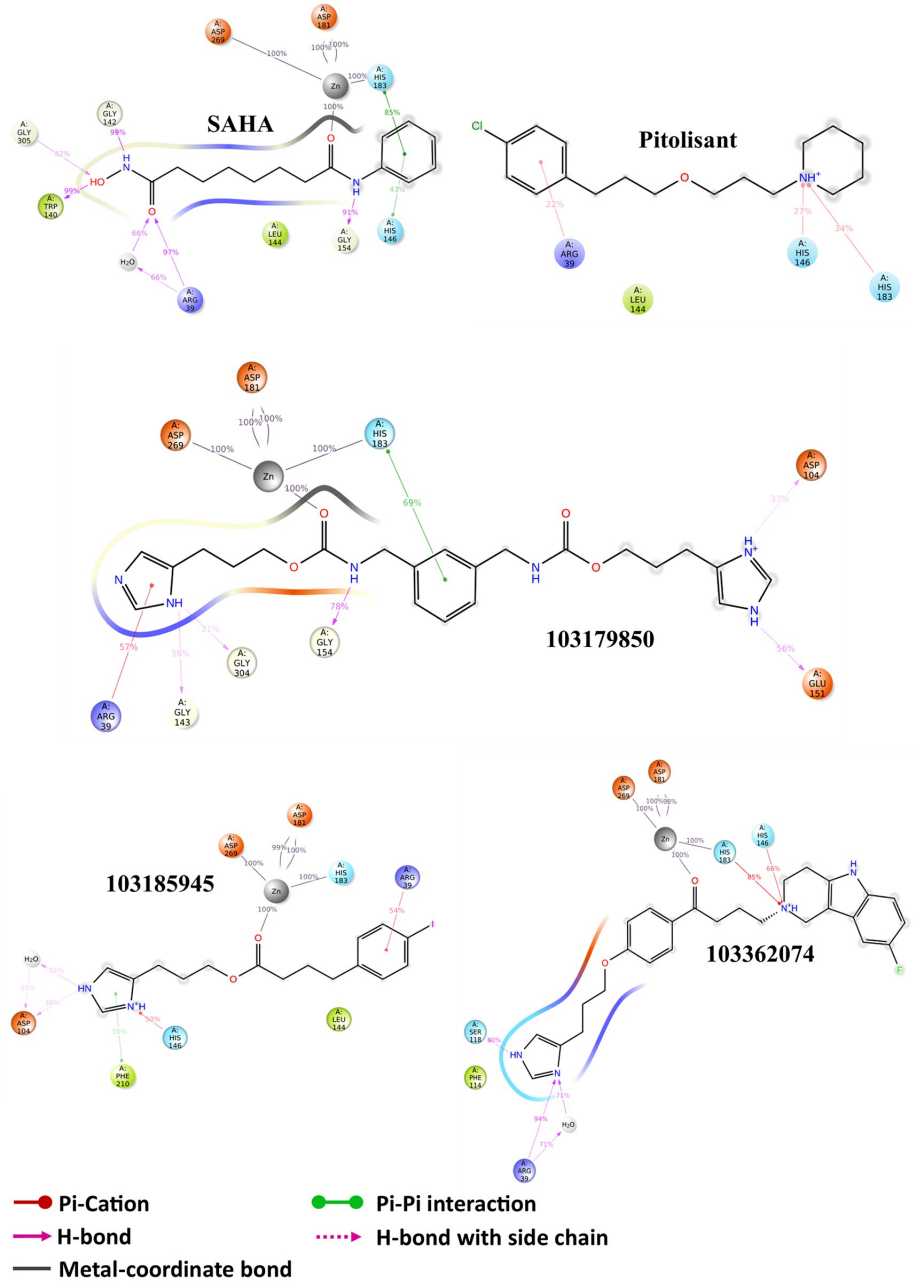
2D interaction diagram of HDAC2 with respective ligands showing the interaction profile of 100ns MD simulation.

To summarize the secondary structure of the HDAC2 binding site, it can be done chiefly in terms of variable loops. Of the eighteen loop regions in one HDAC2 molecule, a total of six variable loops can be surmised to be engaged in the HDAC2 mediated catalysis. The local residue fluctuations of HDAC2 in MD simulation provide valuable insights on the dynamics of these catalytically important variable loops. The protein RMSF in Fig 8B displays the C_α_ fluctuations highlighting the critical loop regions that experience relatively higher variations. Also, the influence of ligand binding is visible in the RMSF plots, especially in the variable loop (VL) 4. Compound 103185945 renders lower fluctuations in VL4 compared to the other two leads, which shoot up to 3 Å and above in the other two cases. Interaction of the above-mentioned leads within this pit of variable loops with facilitated Zn^2+^-mediated metal-coordination governs the effective ligand binding with HDAC2. Hence, special emphasis was given on the binding profile which aligns with the HDAC2 inhibitor pharmacophore features. The schematic 2D protein-ligand interaction diagram averaged on 100ns simulation Fig 9 renders the binding profiles of respective leads in easily interpretable form.

**Fig 10:**
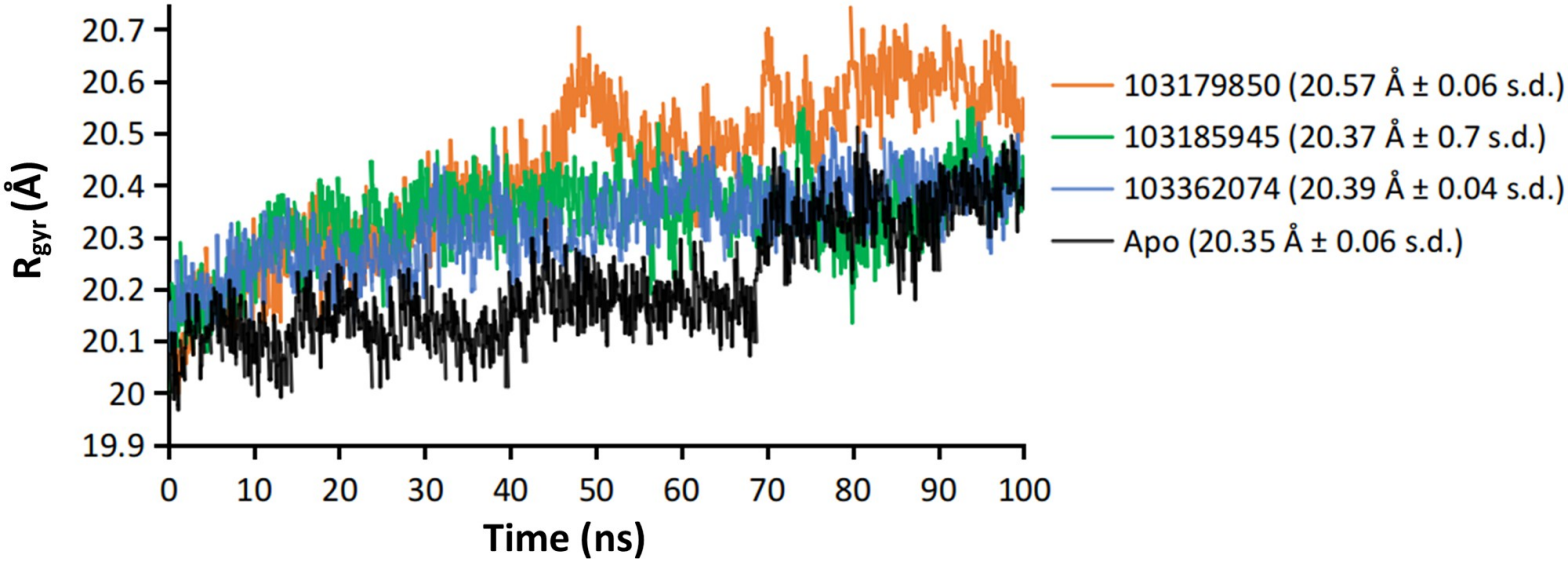
Time series plot of radius of gyration of HDAC2 protein in unbound and ligand-bound form; Average values (in parenthesis) are approximated for relatively equilibrated frames of 60ns and beyond.

While interpreting the MD trajectory data, the inter-atomic distances become one of the most important variables to examine the likelihood of interaction between two molecules [47]. Interatomic distance between the Zn^2+^ atom and the chelating group in the lead molecule with respect to time correlates with the binding site occupancy, owing to the strong metal coordination. Compared to SAHA-bound HDAC2 where the Zn^2+^—O distance was average 2.02 Å with 0.05 Å variation, the averages of Zn^2+^—chelator distance for the leads did not significantly vary, i.e., 2.08 Å ± 0.07 s.d (103179850), 2.15 Å ± 0.08 s.d (103185945) and 2.17 Å ± 0.1 s.d (103362074). These sparingly varying distance values indicate strong metal coordination between the Zn^2+^ and the candidate molecules. As far as the protein’s integrity was concerned, the protein compactness was assessed using the radius of gyration (R_gyr_) of the HDAC2 structure from trajectory frames. Compounds 103185945 and 103362074 retained steadier and slightly higher compaction as compared to 103179850 Fig 10.

### 6. P-L binding free energy converges to steadier values

**Fig 11:**
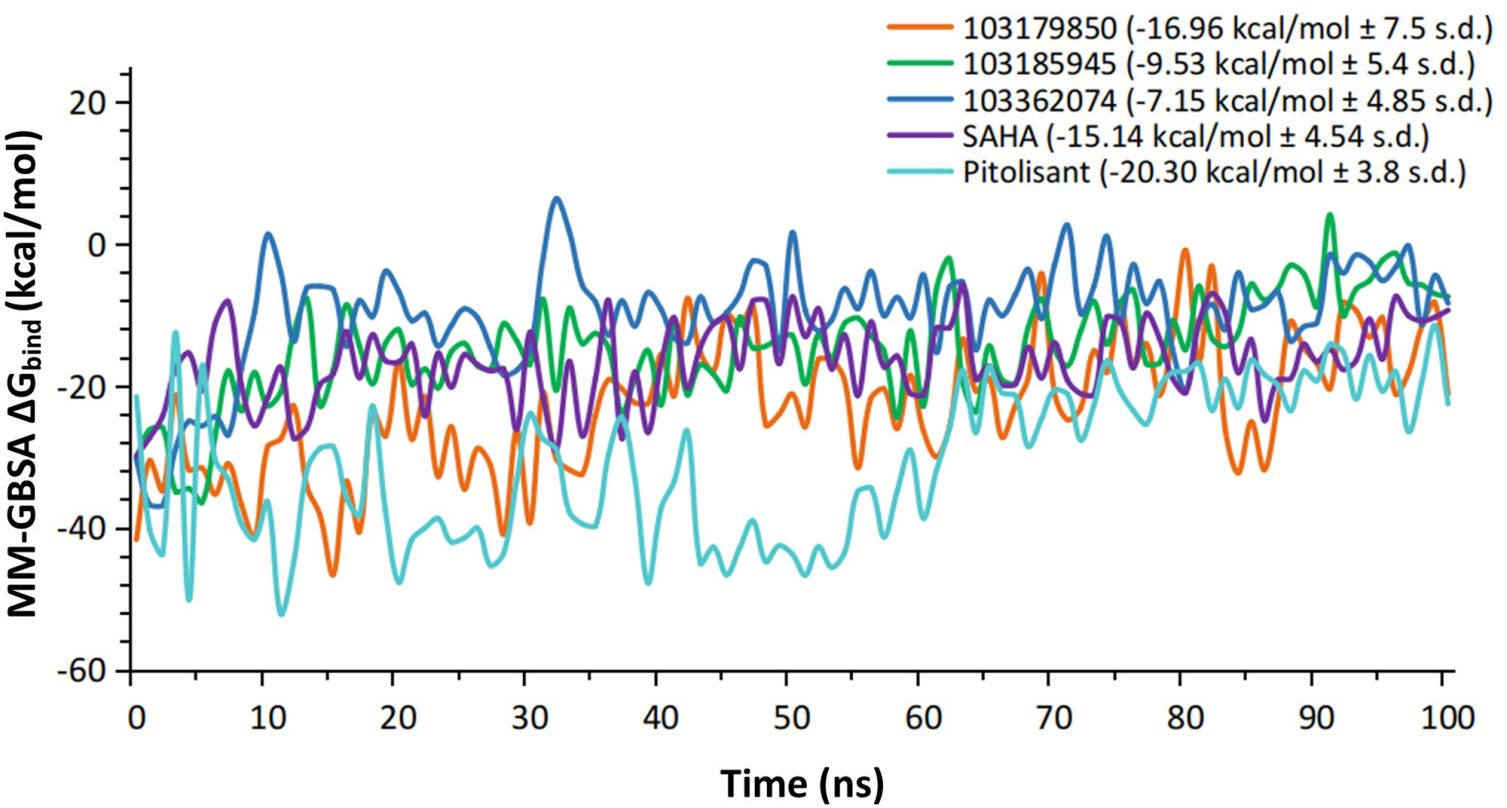
Time series plot of MM-GBSA ΔG_bind_ values of HDAC2 trajectory frames at 1ns interval; Average values (in parenthesis) are approximated for relatively equilibrated frames of 60ns and beyond.

MM-GBSA ΔG_bind_ free energies were calculated for the HDAC2 protein-ligand (P-L) complexes and delineated against simulation time Fig 11. Post-equilibration (~50ns and beyond) analysis of the ΔG_bind_ peaks provides an appropriate comparison of P-L binding and the in-depth observation of the associated trajectory frames suggests that several unanticipated positive values in P-L ΔG_bind_ peaks are attributed to loosely bound Zn^2+^——chelator interactions. The compound 103179850 experiences higher variations in ΔG_bind_ values, while still attaining overall lowerΔG_bind_ values in most frames, averaging for -18.45 kcal/mol, and also retained consistent metal coordination with Zn^2+^. Contrastingly, higher ΔG_bind_ values were observed for 103185945 and 103362074, i.e., an average of -11.05 kcal/mol and -7.63 kcal/mol respectively. Both these compounds underwent loosely bound metal-coordinated states in certain frames nevertheless, experienced lower fluctuations in binding energies. This binding pattern resembles that of SAHA with HDAC2 in that it experiences lower binding free energy variations, suggesting the formation of a steadier P-L complex. In the case of pitolisant, despite its deficient contacts with the Zn^2+^ ion in the HDAC2 inhibition site, pitolisant exhibits overall least energy complex formation (-24.5 kcal/mol), which is solely attributed to its strong hydrophobic interactions with LEU 144, HIS 183, and TYR 308. The Pi-cation contacts of pitolisant’s piperidine ring with HIS 183 infer that it hinders Zn^2+^ ion’s metal coordination with HIS183.

### 7. Computed binding with H3R receptor

**Fig 12:**
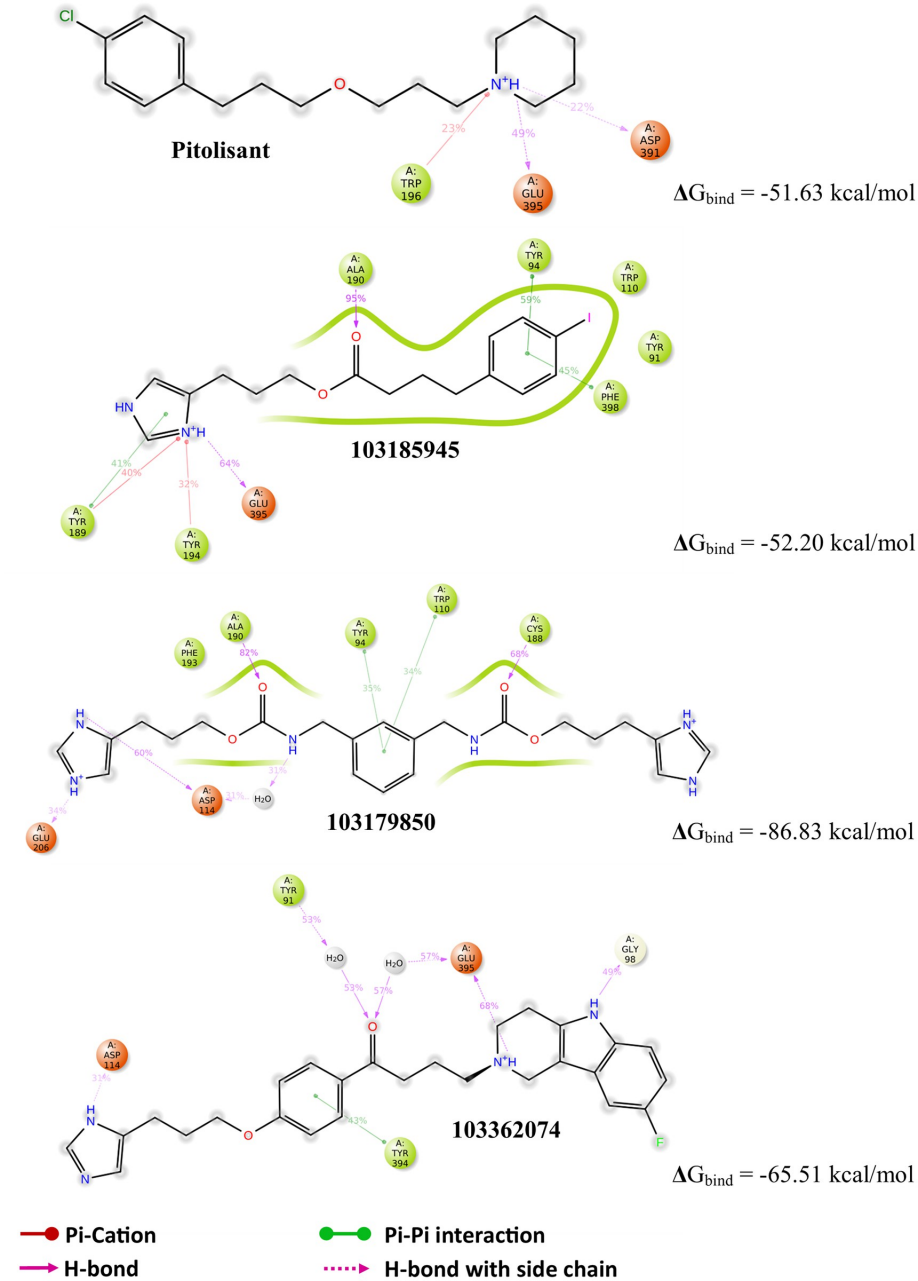
2D interaction diagram of H3R with respective ligands showing the interaction profile of 100ns MD simulation; the MM-GBSA ΔG_bind_ values define the end-point binding free energy.

The fact that the screened leads are pre-analyzed H3R inhibitors questions the specificity of these compounds with respect to pitolisant. Although the experimental Ki value of pitolisant (0.003μM) is significantly lower than 103179850 (0.05 μM), 103185945 (0.041 μM), and 103362074 (0.41 μM), it doesn’t completely account for the molecular affinity and P-L complementarity. The interaction of these three leads and pitolisant with the tRosetta model of H3R Fig 12 distinguishes the enzyme specificity among them as the P-L contacts in the figure show the overall interaction for 100ns of the MD simulation. Note, that the carbonyl groups of these leads are actively involved in H-bond with the H3R active site residues, while the same carbonyl group was involved in metal-coordination with Zn^2+^ in the case of HDAC2. These interactions also highlight the H3R antagonist’s contrasting specificity for Asp 114, Tyr 94, Ala 190, and Glu 395.

### 8. Estimated HDAC2 inhibitory activity of lead molecules

To predict the inhibition potency of lead molecules against HDAC2 based on binding affinity, MM-GBSA of docked lead-HDAC2 complexes were computed [48]. Ganai et al. 2015 suggested that binding free energy(BFE) and IC50 of HDAC2 shows the positive correlation i.e increased negative BFE has lower the IC50(more potent inhibitor) [49]. To approximation of the IC50 of lead molecules was made on the basis of computed MM-GBSA ΔG_bind_ values of reported HDAC2 inhibitors with established IC50 values. A similar trend was found for the calculated MM-GBSA energy with reported IC50 value as also discussed by Bradner et al., 2010 [50] i.e., SAHA (BFE = -28.26 kcal/mol;IC50 = 0.004 μM), Pyroxamide (-18.98 kcal/mol;0.006 μM), CI-994(-10.59 kcal/mol;0.42 μM), HC-Toxin(-9.87 kcal/mol;0.9 μM), Valproate (22.94 kcal/mol; 75 μM) value respectively. Interestingly, the calculated BFE of the leads in the present study exhibit comparatively lower i.e., more negative ΔG_bind_ values within the range of -83 to -62 kcal/mol (Table 3).

**Table 3:** Binding profile with HDAC2 and dual inhibitory fitness of lead molecules.

S no.	(Canonical Smile) Substance SID	GLIDE Docking score	MMGBSA ΔG_bind_ (kcal/mol)	Pharmacophore Fitness score (-1 to 3.0)
**1**	C1 = CC (= CC (= C1)CNC (= O)OCCCC2 = CN = CN2)CNC (= O)OCCCC3 = CN = CN3 103179850	-11.64	-82.42	1.082
**2**	C1 = CC (= CC = C1CCCC (= O)OCCCC2 = CN = CN2)I 103185945	-9.535	-79.26	1.12
**3**	C1CCN(CC1)CCCOC2 = CC = C(C = C2)CN3CCC4 = CC = CC = C4C3 103360761	-6.115	-76.37	2.047
**4**	C1CCN(CC1)CCCOC2 = CC = C(C = C2)CNC3CCC4 = CC = CC = C34 103360711	-4.995	-75.63	1.976
**5**	C1CCC(CC1)CC2 = CC = C(C = C2)OCCCN3CCCCC3 103361521	-8.358	-74.42	1.366
**6**	C1CCN(CC1)CCCOC2 = CC = C(C = C2)CN3CCC(CC3)C (= O)N 103360810	-7.748	-73	1.647
**7**	CN1CCN(CC1)CC2 = CC = C(C = C2)OCCCN3CCCCC3 103360811	-4.099	-72.12	1.608
**8**	C1CCN(CC1)CCCOC2 = CC = C(C = C2)CN3CCOCC3 103360819	-10.442	-70.99	1.178
**9**	C1CN(CC2 = C1NC3 = C2C = C(C = C3)F)CCCC (= O)C4 = CC = C(C = C4)OCCCC5 = CN = CN5 103362074	-7.915	-64.68	1.77
**10**	C1CCC(CC1)NCC2 = CC = C(C = C2)OCCCN3CCCCC3 103360818	-4.621	-62.32	0.874

## Discussion

Metal-dependent histone deacetylases are important epigenetic regulators and are of paramount importance due to their molecular and pharmacological role in critical diseases like cancer, neurodegeneration, and neuropsychiatric disorders [51]. The differential expression of HDAC2 in CNS makes it an alluring therapeutic target for chronic neurological disorders like ones in autism spectrum disorder. In contrast, famotidine (H3R antagonist) has been established to manage autistic-like behavior. Moreover, ASD is medically one of the most enigmatic neurodevelopmental disorders as still has a long way to establish the underlying mechanism. Hence, screening of dual active molecules is one of the novel approaches against ASD. The foundation of this study is based on the notion of identifying potential H3R inhibitors exhibiting high calculated affinity with HDAC2 and acting as a dual-active inhibitor. In order to achieve the aim of the study, we screened FDA approved library and, selected FDA-approved H3R inhibitor molecules that exhibit computationally potent binding on the HDAC2 binding site. Upon thorough literature screening and structural observation, we characterized the binding site of HDAC2 Fig 2A and 2B and important features required for prominent P-L binding. We found that pitolisant, a selective H3R antagonist that is routinely used for the treatment of narcolepsy exhibits the highest affinity for HDAC2 binding site with the embodiment of exemplary HDAC2 pharmacophore, which gave a structural hypothesis for the selection of dual-active inhibitors [52]. Prominent interactions in molecular docking of pitolisant to the HDAC2 are featured by its solvent-exposed piperidine ring, linker region with ether group, and aromatic chlorobenzene group in the foot pocket. We followed a hybrid workflow that included pharmacophore generation using pitolisant, followed by pharmacophore-based and structure-based virtual screening using molecular docking and MD simulation-based P-L binding analyses.

The significance of metal coordinate interaction between the chelating group of ligands, usually ethoxy groups and Zn^2+^ ion was revealed in the case of effective ligand binding in MD simulation. The homogeneity of Zn^2+^——chelator interaction strength was maintained in the case of SAHA and 3 candidate leads i.e., substance id: 103179850, 103185945, and 103362074. It was found that for a lead to attaining stable P-L complex formation with HDAC2, favorable interactions were required with ARG 39 (H-bond) in the foot pocket, Zn^2+^ mediated coordinate interaction with ASP 181, HIS 183 and to some degree with ASP 269 and, decent hydrophobic interaction with the hydrophobic tube of HDAC2, lined mainly by LEU 144 and HIS 146. The important loops were variable loops (VL) 3 and 4, comprising of metal chelator residues and hydrophobic residues respectively. VL-3 and VL-4 accommodate the catalytic site, where the substrate carbonyl is polarized by Zn^2+^ coordination and hydrogen bond, which enables base-promoted nucleophilic attack of a Zn^2+^ -bound water molecule on the substrate carbonyl group [53]. This explains that despite of high structural complementarity of pitolisant with HDAC2, the presence of the ethoxy group doesn’t favor Zn^2+^-mediated polarization. Instead, there are high hydrophobic contacts with the HDAC2 pit residues, LEU 144, PHE 155, and weak H-bonds with ASP 104 at foot pocket. These shortcomings were overcome in the pitolisant-based lead selection, where compounds 103179850, 103185945, and 103362074 contained the carbonyl group which was available for Zn^2+^ coordination. This interaction is in accordance with the inhibitor specificity of HDAC2, validated by the HDAC2-SAHA interaction profile. It was found on closer observation, that compound 103185945 significantly resembles the dynamic attributes shared by SAHA. This is majorly evident from the RMSF time series plot where both demonstrate lower fluctuations of VL-3 and VL-4. The lowering of VL-3 and VL-4 fluctuations corresponds to the minimal deviation of hydrophobic and metal-coordinating residues involved in ligand binding inferring stable P-L complex formation. Analogous results were observed in RMSD, Rgyr, and ΔG_bind_ free energy plots, speculating low fluctuations stabilized contacts.

Recapitulating the principal observations of this study, we state that the three screened H3R inhibitors exhibit promising binding with HDAC2, which might inhibit deacetylation action and hinder the epigenetic control mediated by HDAC2. Compound 103185945 or3-(1H-imidazole-5-yl)propyl 4-(4-iodophenyl)butanoate is synthesized as a novel imidazole derivative and inhibits human histamine H3 receptor at Ki = 0.041 μM. Compound 103179850 or3-(1H-imidazole-5-yl)propyl-N-[[3-[[3-(1H-imidazole-5-yl)propoxycarbonylamino]methyl]phenyl]methyl]carbamate and 103362074 or(*Z*)-but-2-enedioicacid;4-(8-fluoro-1,3,4,5-tetrahydropyrido[4,3-b]indol-2-yl)-1-[4-[3-(1*H*-imidazole-5-yl)propoxy]phenyl]butan-1-one both were tested active inhibitor of histamine H3 receptor in Norway rats, Ki = 0.05 μM and 0.41 μM respectively [54]. Moreover, the results from comparison of the computed ΔG_bind_ values with the reported HDAC2 inhibitors suggest that the selected lead molecules might exhibit lower IC50 values hence pertaining to stronger inhibitory activity against HDAC2. However, a rapid HDAC2 targeted enzyme bioassay might reveal the binding affinity of these three selected compounds *in vitro* with SAHA as the positive control. The inclusion of pitolisant in such a study might highlight and validate the significance of Zn^2+^ in the HDAC2 inhibition mechanism.

### HDAC2, EAAT2 and H3R’s implication in neurological disorders

Animal models of stroke, head trauma, amyotrophic lateral sclerosis (ALS), Alzheimer’s disease, epilepsy, and neurodevelopmental diseases particularly ASD have all shown decreased levels of Glu transporter proteins and/or mRNAs [55–60]. In contrast, individuals suffering from Alzheimer’s and neurodevelopmental disorders(Like ASD) showed the higher HDAC2 level in brain samples. in addition, several animal models of neurological disorders are also evident to have increased HDAC2 levels in brain samples [61, 62]. Moreover, HDAC2 depletion or HDAC inhibitor therapy consistently improves synaptic gene expression, long-term synaptic plasticity, and memory functions, whereas HDAC2 overexpression has the opposite impact [63–67]. Furthermore, The fact that HDACs control EAATs, which have been examined previously, suggests that epigenetic regulation of this family of transporters may be essential in neurological disorders [68]. A group of studies evidenced that, the expression of EAATs is increased by HDAC inhibitors such as trichostatin A and valproic acid (VPA). In astrocytes and oligodendrocytes, VPA raises the level of GLT-1 mRNA and protein [69, 70]. A recently published experiment revealed that HDAC2 suppression enhanced the EAAT2 and VGLUT2 expression following paclitaxel-induced Peripheral Neuropathy [16]. Additionally, H3R inhibition ameliorates the neurobehavioral alteration caused by several neurological disorders [19, 44]. Therefore, the screened dual active leads from the current study could be effectively inhibiting the altered HDAC2 and H3R expression and, eventually could attenuate the neurobehavioral deficit.

## Conclusion

The present study comprehends the application of computational modeling and simulation methods to elucidate the identification of potential HDAC2 inhibitors with predefined histamine H3 receptor antagonistic activity. The prime concept of this study was to computationally examine the specificity of H3R inhibitors against HDAC2 binding sites in search of dual-active inhibitors. It is to be anticipated from the present study that the screened dual-active candidates could manifest the change in epigenetic control of EAAT2 expression carried out by HDAC2 in astrocytic cells. These lead molecules are expected to inhibit the HDAC2 mediated deacetylation of the EAAT2 gene (SLC1A2) and concomitantly inhibit H3R mediated neuroinflammation to become a therapeutic addition in ASD treatment.

## Supporting information

S1 File (DOCX)
