# Supplementary material for: In-silico discovery of dual active molecule to restore synaptic wiring against autism spectrum disorder via HDAC2 and H3R inhibition
Source: PLoS One. 2022 Jul 25;17(7):e0268139. doi: 10.1371/journal.pone.0268139 (PMC9312418; doi:10.1371/journal.pone.0268139)
Supplement: S1 File — (DOCX) [file pone.0268139.s001.docx]

Response:

Thank you so much for accepting the manuscript in your esteemed journal. We have done all the required correction. Please let us know if any

1. Please ensure that the author list and affiliations are correct on the title page of your manuscript, and that your author contributions, competing interests, and financial disclosure are correct as listed below. All of these sections will be indexed in PubMed and published by PLOS ONE as you have written them. Please email plosone@plos.org if any changes to this content need to be made.

   Anupam Raja:
   Conceptualization
   Data curation
   Formal analysis
   Investigation
   Methodology
   Project administration
   Resources
   Software
   Validation
   Visualization
   Writing – original draft
   Writing – review & editing

   Nishant shekhar:
   Data curation
   Formal analysis
   Methodology
   Project administration
   Writing – original draft
   Writing – review & editing

   Harvinder singh:
   Data curation
   Investigation
   Visualization
   Writing – review & editing

   Ajay Prakash:
   Supervision

   Bikash Medhi:
   Investigation
   Supervision
   Writing – review & editing

   Please see here for the full list and definition of contributor roles: http://journals.plos.org/plosone/s/authorship#loc-author-contributions

   Please ensure that the Competing Interests and Financial Disclosure statements listed below are suitable for publication. These sections will be indexed in PubMed and published by PLOS ONE as you have written them. Please email plosone@plos.org if any changes to these statements need to be made.

   Competing Interests: The authors have declared that no competing interests exist.

   Financial Disclosure: We would also like to thank ICMR for providing Anupam Raja with an SRF fellowship only under letter no. 45/30/2020/PHA/BMS.

Response: above all the given information are correct.

2. We note that you have uploaded figures in multiple parts (i.e. Fig 2A, 2B, etc.). Please be advised that PLOS ONE does not publish figures in multiple parts. To ensure that your figures publish correctly, we request that you either separate the multiple parts out into their own figures (i.e. Fig 1A becomes Fig 1, 1B becomes Fig 2, etc.) or that all parts are consolidated into one single figure file, and reupload the files. You can refer to our guidelines for multi-panel figure files here: https://journals.plos.org/plosone/s/figures#loc-multi-panel-figures.

Response: made the change as per the requirement

3. In the text, cite the reference number in square brackets (e.g., “We used the techniques developed by our colleagues [19] to analyze the data”).

Response: made the change as per the requirement

4. To prevent production delays, we recommend using the Author Formatting Checklist to confirm that your paper meets PLOS ONE's typesetting requirements for References, Tables, and Figures: http://journals.plos.org/plosone/s/file?id=c819/plos-one-author-formatting-checklist.docx.

This checklist is a reference tool for you; please do not upload the completed Author Formatting Checklist with your submission files.

Response: made the change as per the requirement

5. To ensure your figures meet our technical requirements, please run each figure included in your submission files through the PACE tool: https://pacev2.apexcovantage.com/. PACE will assess whether your figures meet our technical requirements and will fix the figure(s) or identify any problem(s) that cannot be automatically fixed. It can also convert figures to TIFF format, resize, and rename figures to meet our naming conventions.
To use PACE, first register as a user. Follow the instructions on the site for assessing and converting your figure files. If you experience any difficulty using this tool or have questions about any of the figures and/or images in your paper, please inform the journal office in your response letter.

Response: made the change as per the requirement
